# Acne vulgaris, probiotics and the gut-brain-skin axis - back to the future?

**DOI:** 10.1186/1757-4749-3-1

**Published:** 2011-01-31

**Authors:** Whitney P Bowe, Alan C Logan

**Affiliations:** 1 Department of Dermatology, State University of New York Downstate Medical Center, Brooklyn, New York, 11203, USA; 2 Integrative Care Centre of Toronto, 3600 Ellesmere Road, Unit 4, Toronto, ON M1C 4Y8, Canada

## Abstract

Over 70 years have passed since dermatologists John H. Stokes and Donald M. Pillsbury first proposed a gastrointestinal mechanism for the overlap between depression, anxiety and skin conditions such as acne. Stokes and Pillsbury hypothesized that emotional states might alter the normal intestinal microflora, increase intestinal permeability and contribute to systemic inflammation. Among the remedies advocated by Stokes and Pillsbury were *Lactobacillus acidophilus *cultures. Many aspects of this gut-brain-skin unifying theory have recently been validated. The ability of the gut microbiota and oral probiotics to influence systemic inflammation, oxidative stress, glycemic control, tissue lipid content and even mood itself, may have important implications in acne. The intestinal microflora may also provide a twist to the developing diet and acne research. Here we provide a historical perspective to the contemporary investigations and clinical implications of the gut-brain-skin connection in acne.

## Introduction

The co-morbidity of chronic skin conditions and mental health disorders has long been recognized, and in recent years specialty psychodermatology and neurodermatology groups have emerged. Acne vulgaris is a common dermatological disorder frequently associated with depression, anxiety and other psychological sequelae. The mental health impairment scores among acne patients are higher vs. a number of other chronic, non-psychiatric medical conditions, including epilepsy and diabetes [1-7]. Along with the psychological fallout, there have also been indications that acne patients are at a higher risk for gastrointestinal distress. For example, one study involving over 13,000 adolescents showed that those with acne were more likely to experience gastrointestinal symptoms such as constipation, halitosis, and gastric reflux. In particular, abdominal bloating was 37% more likely to be associated with acne and other seborrheic diseases [8].

The growing awareness that the functional integrity and microbial residents of the intestinal tract may play a mediating role in both skin inflammation and emotional behavior has shed further light on yet another dimension to the relationship between dermatology and mental health. The notion that intestinal microflora, inflammatory skin conditions such as acne, and psychological symptoms such as depression, are all physiologically intertwined is not a new one. Nor is the notion that so-called beneficial bacteria can mediate both skin inflammation and mental health a novel one - what has changed is an accumulation of evidence which provide some early hints at physiological pathways and potential therapeutic avenues in acne vulgaris. Here we review the 70-year-old gut-brain-skin unifying theory, first postulated by dermatologists John H. Stokes and Donald M. Pillsbury in 1930 [9], and provide a historical perspective to the contemporary investigations and clinical implications of the gut-brain-skin connection in acne.

### The Brain-Gut-Skin Theory

Drawing on several lines of experimental evidence and clinical anecdotes, Stokes and Pillsbury provided a 'theoretical and practical consideration of a gastrointestinal mechanism' for ways in which the skin is influenced by emotional and nervous states. These authors connected emotional states - depression, worry and anxiety - to altered gastrointestinal tract function, changes that cause alterations to the microbial flora, which they theorized, in turn promotes local and systemic inflammation. They provided, as they wrote, 'an important linkage of emotion with cutaneous outbreaks of erythema, urticaria and dermatitis by way of the physiology and bacteriology of the gastrointestinal tract'. Citing research showing that as many as 40% of those with acne have hypochlorhydria, Stokes and Pillsbury hypothesized that less than adequate stomach acid would set the stage for migration of bacteria from the colon towards the distal portions of the small intestine, as well as an alteration of normal intestinal microflora. Furthermore, Stokes and Pillsbury suggested that stress-induced alterations to microbial flora could increase the likelihood of intestinal permeability, which in turn sets the stage for systemic and local skin inflammation. The remedies these authors discussed as a means to cut off the stress-induced cycle included the 'direct introduction of acidophil organisms in cultures such as those of *Bacillus acidophilus*'. They also advocated for an acidophilus milk preparation and cod liver oil, long before they would be referred to as probiotics and a rich source of omega-3 fatty acids respectively. Stokes and Pillsbury may well have been aware that some physicians had previously reported mental health benefits with the oral administration of lactic acid bacilli tablets and *Lactobacillus*-fermented drinks [10,11]. Several months before Stokes and Pillsbury completed their theory on the 'emotional linkage' between the brain, gut and skin, another study had reported low stool levels of *L. acidophilus *in 53 patients with a variety of mental health disorders [12]. As we will discuss below, the theory constructed by these authors was well before its time, and indeed much of it, including the potential of probiotics, has subsequently been proven correct in preliminary studies.

### Contemporary Evidence

In recent years it has been confirmed that hypochlorhydria is a significant risk factor for small intestinal bacterial over growth (SIBO). Indeed SIBO is detected via hydrogen breath testing in half of patients on long-term proton pump inhibitor treatment [13]. SIBO presents itself on a wide continuum between being asymptomatic and, at its extreme, a severe malabsorption syndrome. For many, there may be very mild gastrointestinal symptoms, including bloating, diarrhea, abdominal pain, and constipation [14]. It is also reported to be prevalent in functional syndromes such as fibromyalgia and chronic fatigue syndrome [15]. SIBO can compromise proper absorption of proteins, fats, carbohydrates, B vitamins, and other micronutrients due to bacterial interference. Excess bacteria can successfully compete for nutrients, produce toxic metabolites, and cause direct injury to enterocytes in the small intestine [16]. Just as Stokes and Pillsbury had supposed, SIBO has recently been shown to be associated with increased intestinal permeability, whereas antimicrobial treatment of SIBO helps to restore the normal intestinal barrier [17]. Experimental studies show that psychological stress stagnates normal small intestinal transit time, encourages overgrowth of bacteria, and compromises the intestinal barrier [18]. SIBO is strongly associated with depression and anxiety, while eradication of SIBO improves emotional symptoms [19,20]. Although the frequency of SIBO in acne vulgaris has not yet been investigated, a recent report indicates that SIBO is 10 times more prevalent in those with acne rosacea vs. healthy controls. Correction of SIBO leads to marked clinical improvement in patients with rosacea [21]. The oral administration of probiotics has also proven beneficial in the reduction of SIBO [22]. Interestingly, the omega-3 fatty acid-rich cod liver oil advocated by Stokes and Pillsbury may have been ahead of its scientific time. Not only does an omega-3 deficient diet increase SIBO [23], it has also been linked multiple times to an increased risk of depressive symptoms [24]. A small series of case reports indicates value of omega-3 fatty acids in both the clinical grade of acne and global aspects of well-being [25].

As for intestinal permeability in acne vulgaris, there have been hints that the intestinal lining may be compromised. One older study used a blood serum complement fixation test and reported that acne patients were more likely to show enhanced reactivity to bacterial strains isolated from stool. Approximately 66% of the 57 patients with acne showed positive reactivity to stool-isolated coliforms, this compared to none of the control patients without active skin disease [26]. Furthermore, a study involving 40 acne patients showed both the presence of, and high reactivity to, lipopolysaccharide (LPS) endotoxins in the blood as measured by the stellate fibrin crystal test. None of the matched healthy controls reacted to the *E. coli *lipopolysaccharide endotoxin (*E. coli *LPS), while 65% of the acne patients did have a positive reaction [27]. The inference of these results is that circulating endotoxins derived from gut microbes is not an uncommon feature of acne vulgaris, and one indicating that intestinal permeability is a potential issue for a sizable group of acne patients. Since systemic *E. coli *LPS itself can produce depression-like behavior in animals [28], and enhanced reactivity to *E.coli *LPS is noted in irritable bowel patients with higher anxiety levels [29], an updated investigation in acne seems warranted.

At this point it is unknown if, as confidently suspected by Stokes and many of his contemporaries, constipation is more prevalent in those with acne. They considered it to be an 'important factor' [30] and even 'the rule' rather than the exception [31]. One older study using a bismuth test beverage and objective fluoroscopy did report intestinal stagnation in 47% of a small group of acne (n = 30) acne patients. They also reported constipation as a clinical complaint in 40% of acne patients [32]. Even if constipation were more frequent, as the recent population study involving 13,000 adolescents indicates [8], it would be tempting to dismiss it as having no relevance whatsoever to the pathogenesis of acne and/or depression. Yet, an important study in 2005 should provide cause for further consideration; among 57 patients with functional constipation, fecal concentrations of *Lactobacillus *and *Bifidobacterium *were significantly lower and intestinal permeability was significantly higher compared to healthy adults without constipation. In addition, there was an enhanced systemic immune response, almost certainly due to larger molecules gaining access across the intestinal barrier [33]. Separate research has recently shown that chronic constipation (in otherwise healthy adults without irritable bowel syndrome) is associated with marked alterations to the intestinal microflora [34]. Combined with new findings indicating increased gut permeability in those with depression [35], we must surely reframe the obvious overlap between depression and constipation [36], and the more specific finding of longer whole gut transit time positively correlated with depression [37].

### Intestinal Microflora

The Stokes-Pillsbury theory was also predicated upon changes not only to the residential location of microbes within the intestinal tract, they suspected that a quantitative alteration to the microrobial flora was also at play. This suggestion has also been supported by contemporary investigations. Experimental and human studies have shown that a variety of psychological and physiological stressors - confinement, extremes of temperature, crowding, acoustic, academic examination - can impair normal intestinal microflora [38-40]. Most notable are stress-induced reductions in *Lactobacillus *and *Bifidobacteria *species.

The potential of stress-induced changes to the gastrointestinal microflora among acne patients has sadly received little attention. The first attempt to determine if there were differences in the intestinal bacterial microflora was a 1955 investigation that focused on the presence or absence of potentially pathogenic bacteria in 10 acne patients vs. controls. Obviously there can be few generalizations drawn from a study involving only 10 subjects with acne, and the authors simply concluded that there appeared to be no major differences (vs. controls) in a small selection of enteric bacteria genera under culture technique [41]. However, we find it noteworthy that *Bacteroides *spp were more commonly isolated from the acne patients, particularly since elevations of *Bacteroides *have been noted in humans under psychological stress [42]. Unfortunately this pilot investigation was restricted to a small group of bacterial genera and did not culture for potentially beneficial bacteria such as *Lactobacilli *and *Bifidobacterium*. The only other investigations examining the intestinal microflora in acne, to our knowledge, are within non-English language journals. A Russian investigation reported that 54% of acne patients have marked alterations to the intestinal microflora [43], while a Chinese study involving patients with seborrheic dermatitis also noted disruptions of the normal gastrointestinal microflora [44]. With recent advances in molecular identification of intestinal microbial inhabitants, we are hopeful that investigators will take a renewed interest in potential changes to the enteric microbial profile among acne patients.

### Probiotic Administration

As mentioned, Stokes and Pillsbury made numerous references to the use of *L. acidophilus *and *L. acidophilus*-fermented milk products as a treatment modality in the context of the brain-gut-skin inflammatory process. Indeed, other physicians writing in the 1930s made reference to the popularity of *L. acidophilus *cultures among the general public as an internal means to treat acne [45]. However, despite the apparent appeal of what would later be described as probiotics, there was little research to determine efficacy. The first formal clinical case report series on the potential value of *Lactobacillus *probiotics was published in 1961. A physician from Union Memorial Hospital in Baltimore, Robert H. Siver, followed 300 patients who were administered a commercially available probiotic (Laxtinex tablets providing a mixture of *L. acidophilus *and *L. bulgaricus*). He used a protocol of probiotic supplementation for 8 days followed by two-week wash out then re-introduction for an additional eight days. The rationale for such a dosing regimen is unclear. In any case, he reported that 80 percent of those with acne had some degree of clinical improvement, and that the intervention was most valuable in cases of inflammatory acne. Without a placebo control, Dr Siver concluded merely that 'interactions of skin manifestations of acne vulgaris and of metabolic processes of the intestinal tract are suggestive' [46].

More recent investigations involving the internal application of probiotic supplements in acne are restricted to non-English language journals. The first, an Italian study involving 40 patients, added an oral supplement of 250 mg freeze-dried *L. acidophilus *and *B. bifidum *as an adjuvant to standard care in half the group. In addition to better clinical outcomes among the patients supplemented with probiotics, the researchers reported better tolerance and compliance with antibiotics [47]. Additional research from Russia supports the benefit of probiotics added to standard care, with a reported acceleration in time to significant clinical improvement in those who had been administered probiotics [43]. It is difficult to critically evaluate these foreign investigations, for now they should serve as simply as a further indication that oral probiotics warrant thorough investigation in acne vulgaris. In the meantime, a recent study involving 56 patients with acne showed that the consumption of a *Lactobacillus*-fermented dairy beverage improved clinical aspects of acne over 12 weeks. Specifically, the probiotic drink consumption led to significant reductions in total lesion count in association with a marked reduction in sebum production. Although the addition of lactoferrin (an anti-inflammatory milk protein) to the probiotic drink did provide greater efficacy in the reduction of inflammatory lesions, the benefits of the probiotic drink alone lend further support to the notion that probiotics have an adjuvant role to play in acne therapy [48].

The theoretical value of oral probiotics as adjuvant care in acne vulgaris seems sound. Recent studies have shown that orally consumed pre and probiotics can reduce systemic markers of inflammation and oxidative stress [49-51]. Since the local burden of lipid peroxidation in acne is high, such that it appears to place a great demand upon blood-derived antioxidants [52], the ability of oral probiotics to limit systemic oxidative stress [53] may be an important therapeutic pathway. Oral probiotics can regulate the release of inflammatory cytokines within the skin [54], and a specific reduction in interleukin-1 alpha (IL-1-α), noted under certain experimental conditions [55], would certainly be of potential benefit in acne. In line with observations of internal antibiotic use, it is also true that oral encapsulated probiotics have the potential to change the microbial community at sites far removed from the gastrointestinal tract [56]. We will address the potential of probiotics to mediate acne through the gut-brain connection shortly.

### Topical Probiotics

While the intent of this review is to focus on the brain-gut-skin connection, the ability of ingested probiotics to alter distant microbial residents suggests that topical probiotics are worthy of brief discussion. Once again, the notion that topically applied probiotics may be helpful in acne vulgaris is not a new one. The first report that 'topical bacteriotherapy' (via local *Lactobacillus bulgaricus *application) may be helpful in acne and seborrhea was published in 1912 [57]. However, it was not until 1999 that proper scientific technique was used to evaluate some of the potential skin-specific benefits of lactic acid bacterial application. Specifically, researchers showed that the lactic acid bacteria *Streptococcus thermophilus*, a species found in most yogurts, can increase ceramide production when applied to the skin for 7 days as a cream [58]. This work, which has since been replicated [59,60], is of relevance to acne, particularly when considering that some of the ceramide sphingolipids, most notably phytosphingosine (PS), provide both antimicrobial activity against *Propionibacterium acnes *(*P. acnes*) and direct anti-inflammatory activity [61]. Sphingolipids have been noted to be low in acne [62], and the seasonal loss of ceramides may be a driving force behind much higher dermatological office visits for acne during winter months [63]. Indeed, topical application of 0.2% PS reduced papules and pustules by 89% in a recent 2-month pilot study [61]. Additional studies hinting at the value of topical probiotics in acne include recent reports that strains of *Bifidobacterium longum *and *Lactobacillus paracasei *can attenuate skin inflammation mediated by substance P [64,65]. This is of relevance because substance P may be a primary mediator of stress-induced amplification of inflammation and sebum production in acne [66]. Two separate reports have also indicated that various probiotic lactic acid bacteria can provide *in vitro *antimicrobial activity against *P. acnes *[67,68]. The latter study also used a clinical arm, the results showing that topical application of an *Enterococcus faecalis *probiotic lotion for 8 weeks reduced inflammatory lesions by over 50% vs. placebo [68]. Certain substances secreted by bacterial strains, such as antimicrobial peptides, have been shown to inhibit growth of P. acnes. *Streptococcus salivarius*, a prominent member of the oral microbiota of healthy humans, has been shown to secrete a bacteriocin-like inhibitory substance (BLIS-like substance) capable of inhibiting *P. acnes *[69]. In addition to the antimicrobial activity, *S. salivarius *bacterial cells themselves inhibit a number of inflammatory pathways, thus acting as immune modulators [70]. Finally, the application of select bacteria to the skin may provide a protective shield, similar to a physical barrier. This so-called bacterial interference, through competitive inhibition of binding sites, is thought to prevent colonization by other, potentially pathogenic, bacterial strains [71].

### Internal Bacteriotherapy and The Gut-Brain-Skin Triangle

As mentioned, there had been older commentaries and clinical anecdotes suggesting that orally consumed lactic acid bacteria might be of benefit in alleviating depressive symptoms. It was also reported that patients with mental health disorders appeared to have very low levels of *L. acidophilus*. In a series of case reports in 1924, one Illinois physician reported value of oral *L. acidophilus *for the treatment of both acne and mental health disorders; in addition to L. acidophilus improving the complexion, it was stated that 'in certain patients it even seemingly contributes to mental improvement' [72]. It was also reported that the yeast *Saccharomyces cerevisiae *could improve both acne vulgaris and constipation when orally consumed [73], an interesting anecdote when considering new research on the ability of this organism to improve the integrity of a disturbed gut barrier [74]. However, these early observations were mostly explained as simply the ability of administered lactic acid bacteria to improve bowel function and reduce constipation. Too often clinicians in the early part of the 20^th ^century associated constipation with the root of all acne and depression. These clinical case reports were never advanced to proper scientific investigations.

In the last decade, following the publication of two influential hypotheses papers [38,39], the potential physiological mechanisms by which mental health might be influenced by intentional manipulation of the intestinal flora have finally been explored. The oral administration of probiotics via laboratory chow has been shown to increase peripheral tryptophan levels as well as alter serotonin and dopamine turnover in the frontal cortex and limbic system [75]. Indeed, oral probiotics appear to increase resiliency of nerve cells and reduce apoptosis during conditions of experimental physiological stress [76]. The addition of probiotic to laboratory chow increases the tissue levels of omega-3 fatty acids necessary for normal mood states [77], while in humans the plasma levels of anti-inflammatory fatty acids increases when co-administered with probiotics [78]. The presence in the gastrointestinal tract of non-pathogenic bacteria such as *Bifidobacteria *appear to attenuate an exaggerated stress response, and maintain levels of brain derived neurotrophic factor (BDNF), a neuropeptide known to be low in depression [79]. On the other hand, even mild degrees of chronic gastrointestinal tract inflammation can provoke anxiety and diminish BDNF production in animals [80]. Recently it was reported that in addition to systemic protection against lipid peroxidation, oral Bifidobacterium decreased brain monoamine oxidase activity, thereby potentially increasing neurotransmitter levels between synapses [81]. In experimental models of psychological stress, oral *Bifidobacteria *reduces systemic inflammatory cytokines and normalizes brain levels of stress hormones in rats, while intentionally manipulating the diet of animals such as to double the fecal *Lactobacillus *counts, results in decreased anxiety-like behavior [82,83].

The influence of probiotics as a means to attenuate substance P release, both in the intestinal tract and the skin [64,84], cannot be overlooked as a relevant pathway connecting the nervous system to the gut and the skin. Experimental alterations to the normal gut microbiota can increase substance P release in the nervous system and promote behaviors reflective of anxiety [85]. Indeed, even minute elevations in circulating substance P can lead to anxiety, depression and aggression [86]. Conversely, those who respond to antidepressant pharmacotherapy are known to have declines in serum substance P in conjunction with improved mood states [87]. Thirty years have passed since it was discovered that biologically active peptides such as substance P not only communicate within the gut, brain and skin, they are also of common embryonic origin [88]. With emerging research showing that substance P increases sebum production, surely this pathway warrants serious investigation.

**Figure 1:**
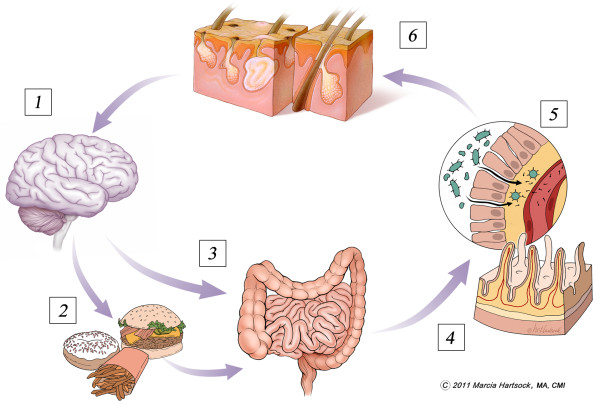
**Potential Pathways of the Gut-Brain-Skin Axis in Acne Vulgaris**: [1] Psychological distress alone or in combination with [2] high fat diet, processed comfort foods devoid of fiber, cause alterations to [3] gut motility and microbiota profile [4]. Loss of normal microbial biofilm (*Bifidobacterium *in particular) causes intestinal permeability and endotoxins gain systemic access [5]. Burden of inflammation and oxidative stress is increased, substance P is elevated, insulin sensitivity is decreased due to endotoxemia [6]. In those genetically susceptible to acne vulgaris, this cascade increases the likelihood of excess sebum production, exacerbations in acne and additional psychological distress. Both probiotics and antimicrobials may play a role in cutting off this cycle at the gut level.

An additional mechanism whereby probiotics might influence both mood and acne is via regulation of glycemic control. In recent years it has become evident that there may indeed be a connection between dietary components, most notably low-fiber carbohydrates, and the risk of acne [89]. For example, regional diets low in processed foods and sugars (with an overall low glycemic load) are associated with decreased acne risk. Intervention studies using similar low glycemic load meals have reported improvements [90,91]. On the other hand, even in healthy adults, epidemiological studies have made associations between blood chemistry indicative of insulin resistance and an elevated risk of depressive symptoms [92,93]. This is of relevance because emerging research shows that the gut microbiota contributes to glucose tolerance [94], and that orally administered *Bifidobacterium lactis *can improve fasting insulin levels and glucose turnover rates, even in the presence of a high-fat diet [95]. While much more research is necessary, the mechanisms appear to involve the ability of *bifidobacteria *to prevent the efflux of lipopolysaccharide (LPS) endotoxins into systemic circulation. Specifically, the loss of *bifidobacteria *by poor dietary choices - high fat, sugar - leads to increased intestinal permeability, encroachment of LPS endotoxins through the intestinal barrier, which in turn leads to low-grade inflammation, oxidative stress, insulin resistance and sickness behavior [96,97]. In humans, probiotic administration may diminish systemic access of gut-derived LPS endotoxins and reduce reactivity to such endotoxins [98]. This entire picture takes on greater meaning when considering recent international studies showing that acne is associated with increased consumption of highly palatable, sweet, fried, calorie-rich foods with low nutrient density [99-101] - and that it is well documented that a period of insulin resistance occurs during puberty [102], one coinciding with the development of acne, depression and/or anxiety. Therefore, it seems reasonable to ask, to what degree might the gut microbiota influence these processes and disease risk during puberty? A summary of potential pathways of interaction between the brain-gut-skin axis in acne is provided in figure 1.

We also find it noteworthy that of three large population studies linking dairy consumption (most notably milk) and acne, none made a positive correlation between fermented dairy (e.g. yogurt) and acne [103-105]. It has been postulated that milk is associated with acne because it contains growth hormones (both synthetically added and naturally occurring) [106]. Acne is certainly driven by insulin-like growth factor I (IGF-I) [107], and IGF-I can be absorbed across colonic tissue [108]. Therefore, it is interesting to note that probiotic bacteria (*Lactobacilli *in particular) utilize IGF-I during the fermentation process when added to milk, with a resultant 4-fold lower level of IGF-I in fermented vs. skim milk [109]. If, as we suspect, there is increased intestinal permeability in acne, the intestinal absorption of IGF-I would likely be enhanced in general, and more specifically, when milk (rather than fermented dairy) is orally consumed. In sum, researchers may need to look more closely at why fermented milk/dairy has escaped association with acne, while other forms of dairy have not.

### Intervention Studies

The first formal investigation of the potential psychological benefits of probiotic supplementation in humans involved 132 otherwise healthy adults; those who had more depressive sympotoms at baseline had significant improvement in mood scores after taking a probiotic *Lactobacillus casei *fermented beverage compared to the placebo group [110]. A separate placebo-controlled study involved 39 chronic fatigue syndrome patients who were administered the same oral *Lactobacillus casei *probiotic vs. placebo. At the conclusion of the 8-week study, depression scores remained unchanged between the groups, however there were significant improvements in anxiety as measured via the Beck Anxiety Inventory vs. placebo [111].

Even more recently, French researchers evaluated a *Lactobacillus helveticus *and *Bifidobacterium longum *combination probiotic which was orally administered for one month in a placebo-controlled study. Using a variety of validated anxiety, stress, and depression scales, researchers reported significant improvements in day-to-day depression, anger, anxiety, as well as lower levels of the stress hormone cortisol among otherwise healthy adults taking a daily probiotic supplement vs. placebo. In addition, an experimental arm of this study also confirmed that the probiotic added to the chow of rats did indeed decrease behaviors indicative of anxiety [112]. In a study involving 44 patients with irritable bowel syndrome, the oral consumption of a prebiotic fiber (trans-galactooligosaccharide) significantly reduced anxiety in conjunction with the expected marked elevations in fecal *bifidobacteria *levels [113]. Finally, research has also shown that the administration of the soil-based organism *Mycobacterium vaccae *can significantly improve quality of life, depression and anxiety (vs. control) in patients receiving chemotherapy for lung cancer [114].

Surely the publication of these studies must allow us to consider the possibility that the psychological impairment in acne could be, at least to some degree, mediated by endogenous factors that include the gut microbiota. Over the years some researchers and clinicians have suggested the existence of an 'acne personality', one that predates the disease onset and subsequently increases the likelihood of stress reactivity, anxiety and depression associated with acne [115-119]. Since most investigations have looked at the post-acne psychological impairments, a largely justifiable view is that the risk of anxiety and depression is strictly associated with a disease that presents itself so visibly in most cases. It is not our position to infer that this view is incorrect; rather we contend that endogenous factors may also play a mediating role in the elevated risk. We must ask why is it that despite marked clinical success with topical and oral interventions, a number of studies using validated measurements of depression, mood and quality of life, indicate that the mental outlook remains unchanged [120-123]? Indeed, in one of the studies cited [120], mood scores declined despite significant clinical improvement with topical interventions. In a systematic review examining depression and isotretinoin, only 1 out of 4 studies using validated depression instruments showed a statistically significant reduction in depressive symptoms [124]. It seems remarkable that an agent with such obvious clinical benefit would only show a trend toward improving depressive symptoms and mental outlook.

### Future Directions

It seems obvious that we can no longer offhandedly dismiss a potential relationship between the GI microflora, mental health and acne vulgaris. Just a few short years ago, the suggestion that the gut microbiota might be a significant factor in the development of obesity seemed obscure. Yet, in the last 48 months, a growing body of research is underscoring a very significant relationship between gut microflora, systemic low-grade inflammation, metabolism, blood lipids and fat storage [125,126]. At present, there are many questions that require resolution. Are the regional differences in acne, for now linked to a high fiber, low glycemic load diet, in any way connected to the relationship between such diets and the intestinal microflora? Rates of acne have been documented to be extremely low in isolated hunter-gatherer communities [127] - dietary and lifestyle habits in these locations would almost certainly alter intestinal microflora directly via root fiber and also bring individuals into greater contact with a variety of soil-based organisms. We know that the typical Western diet, high in sugar and fat, devoid of fiber, the very one correlated with risk of acne, is associated with lower levels of *Lactobacillus *and *Bifidobacterium *[128-131]. The mechanism(s) of action of oral antibiotics in acne remains a mystery - is it a systemic effect against *P. acnes*, an anti-inflammatory influence, ability to lower sebum free fatty acids, or via antioxidant activities [132,133]? Could it be due to the influence of antibiotics on the gut microbiota, which in turn improves glycemic control and decreases LPS endotoxin encroachment into the periphery [134]? Is there a sizeable sub-population of acne patients wherein intestinal permeability and SIBO are playing a contributing role, connecting acne itself with a predetermined higher risk of depression and anxiety? In other words, might the gut-brain-skin triangle play even a small role in the much higher rates of depression and anxiety in acne? We suspect that it does. As we discussed, the gut microbiota influences systemic lipids and tissue fatty acid profiles; therefore it is reasonable to ask if it might influence overall sebum production and specific free fatty acids within sebum. It would be interesting to determine if oral rifaximin, an antibiotic which has no systemic antimicrobial activity, is effective in acne vulgaris, mood and quality of life.

Moving forward we must attempt to answer these and many other plausible questions. Acne vulgaris is a complex disease without a single avenue of pathogenesis, therefore scientists and clinicians must remain open-minded to unexpected therapeutic pathways. To date, Stokes, Pillsbury and other dermatology elders have been validated in many aspects. However, we cannot rely on anecdotes, inferences and uncontrolled observations; we must approach this hypothetical landscape, the gut-brain-skin triangle, with scientific vigor.

## Conclusion

The scientist and philosopher Goethe is quoted as saying 'everything has been thought of before, but the difficulty is to think of it again'. Based on our review of the original hypotheses of Stokes, Pillsbury and their peers, it seems much of the recent scientific endeavors in the area of the gut-brain-skin axis, in the broad sense, have been thought of before. The difference, of course, is the degree of scientific sophistication with which we can now see an undeniable link between these major organ systems. The lines of communication, as mediated by gut microbes, may be direct and indirect - ultimately influencing the degree of acne by a systemic effect on inflammation, oxidative stress, glycemic control, tissue lipid levels, pathogenic bacteria, as well as levels of neuropeptides and mood-regulating neurotransmitters. It was not the contention of Stokes and Pillsbury, nor is it ours, that acne is a disease of the gastrointestinal tract. Yet, there appears to be more than enough supportive evidence to suggest that gut microbes, and the integrity of the gastrointestinal tract itself, are contributing factors in the acne process. Only well designed trials can determine what, if any, the degree of contribution might be.

## Competing interests

The authors declare that they have no competing interests.

## Authors' contributions

WPB and ACL contributed equal time and effort in the investigation, research and drafting of this manuscript. All authors read and approved the final manuscript.
